# Detection of referable diabetic retinopathy using machine learning on routine clinical data

**DOI:** 10.3389/fmed.2026.1807809

**Published:** 2026-05-08

**Authors:** Young Joon Jeon, Jae Shin Song, Shubham Borghare, Youngju Lee, Young Wook Choi, Junghan Song, Soo Lim, Se Joon Woo

**Affiliations:** 1 Department of Ophthalmology, Seoul National University College of Medicine, Seoul National University Bundang Hospital, Seongnam, Republic of Korea; 2 RetiMark R&D Center, Seoul, Republic of Korea; 3 Department of Laboratory Medicine, Seoul National University College of Medicine, Seoul National University Bundang Hospital, Seongnam, Republic of Korea; 4 Department of Internal Medicine, Seoul National University College of Medicine, Seoul National University Bundang Hospital, Seongnam, Republic of Korea

**Keywords:** artificial intelligence, clinical decision support system, diabetic retinopathy, machine learning, random forest

## Abstract

**Background:**

Early detection of referable diabetic retinopathy (RDR) is crucial to prevent vision loss. We developed and validated a machine learning (ML) model using clinical and laboratory variables to predict RDR without ophthalmic imaging.

**Methods:**

We enrolled 562 adults with diabetes who underwent fundus examination at a single tertiary center from June 2015 to December 2023, retrospectively and prospectively. RDR was defined as moderate nonproliferative diabetic retinopathy or worse, or diabetic macular edema. Predictors included demographic factors, diabetes duration, glycemic control, blood pressure, lipid profiles, and kidney function markers. Patients were randomly divided into training (*n* = 175) and validation (*n* = 387) sets. Four ML models were trained, and performance was evaluated using the area under the receiver operating characteristic curve (AUROC). Predictor importance was assessed using Shapley Additive Explanations (SHAP).

**Results:**

In the validation set, the random forest achieved the highest performance, with an AUROC of 0.932 (95% confidence interval, 0.90–0.96), sensitivity of 85.8%, specificity of 91.2%, and accuracy of 87.9%. SHAP ranked 15 predictors, with age showing the highest importance, followed by diabetes duration, fasting glucose, body mass index, diastolic blood pressure, height, smoking history, Cystatin C, systolic blood pressure, hemoglobin A1c, weight, estimated glomerular filtration rate, total cholesterol, insulin use, and sex.

**Conclusion:**

A random forest model using routinely available clinical data identified RDR without fundus imaging. It may serve as a practical tool for early detection of RDR in resource-limited settings, enabling timely referral and supporting integration into clinical decision support systems.

## Introduction

Diabetic retinopathy (DR) is a leading cause of vision loss and a common microvascular complication of diabetes mellitus (DM) (1). With the global rise in diabetes (2), early detection of DR is essential to prevent progression and preserve vision (3, 4). Thus, distinguishing referable diabetic retinopathy (RDR) from non-RDR is crucial for developing cost-effective early screening guidelines.

Although artificial intelligence (AI) systems using fundus photography or optical coherence tomography (OCT) have shown excellent performance in DR detection (5, 6), their reliance on imaging infrastructure limits accessibility, especially in low-resource environments. In contrast, clinical and laboratory data are routinely available but under-utilized in DR screening. Prior studies using such data have shown modest performance, highlighting the need for improved models.

In this study, we developed and validated a machine learning (ML) model using only systemic clinical data to detect RDR, aiming to provide an accessible and interpretable screening alternative. We further implemented the model into a clinical decision support system (CDSS) to facilitate automated risk assessment and referral support.

## Methods

### Study design and setting

We conducted a hybrid observational study including both prospective and retrospective cohorts at Seoul National University Bundang Hospital (SNUBH). The prospective cohort included 400 patients treated for diabetes in the Department of Endocrinology between June 2022 and December 2023. The retrospective cohort included 300 patients with prior DR diagnosis who had consented to a secondary genetic study between June 2015 and December 2023. Each consenting participant was assigned a unique case identifier to ensure de-identification and protect confidentiality.

The study was approved by the institutional review board of SNUBH (IRB No. E-2201-732-350, B-0912-089-011) and the study was carried out in accordance with the tenets of the Declaration of Helsinki.

### Participants

Inclusion criteria were age 20–89 years, type 1 or type 2 diabetes duration ≥ 3 years, and available fundus imaging. Patients were excluded if they had recent ophthalmic treatment, glycosylated hemoglobin (HbA_1c_) ≥ 143 mmol/mol (13%), or had received ophthalmic treatments for DR (including laser therapy, intravitreal injections, or surgery) within the preceding 6 months.

Participants unable to undergo the required blood sampling or those whose retinal imaging was insufficient for accurately diagnosing and staging DR were also excluded. All eligible individuals received detailed information about the study and provided informed in the prospective cohort consent.

### Data collection

Clinical information obtained for each participant included demographic characteristics (age, sex), diabetes-related factors (duration of diabetes, diabetes type, family history of diabetes, history of oral hypoglycemic agents or insulin use), lifestyle variables (smoking status), comorbidities (diabetic nephropathy, neuropathy, cardiovascular disease, cerebrovascular disease, hypertension, and dyslipidemia), and physical measurements (height, weight). Additional clinical parameters included vital signs [systolic blood pressure (SBP), diastolic blood pressure (DBP), pulse rate] and laboratory findings such as blood cell counts, hemoglobin, hematocrit, platelets, fasting glucose, HbA_1c_, serum albumin, creatinine, Cystatin C, and estimated glomerular filtration rate (eGFR), which was calculated using the Chronic Kidney Disease Epidemiology Collaboration (CKD-EPI) formula (7). Lipid profiles (total cholesterol, triglycerides, high-density lipoprotein, and low-density lipoprotein), liver enzymes (aspartate aminotransferase, alanine aminotransferase), C-peptide, C-reactive protein, and urinary markers (protein–creatinine ratio, microalbumin–creatinine ratio, and counts of red and white blood cells) were also obtained.

DR was assessed by Optos ultrawide field fundus photography (Optos plc, Dunfermline, Scotland, UK), with a single image centered on the fovea for each eye. One ophthalmologist with more than 10 years of experience in grading DR, certified and accredited in ophthalmological diagnostics, reviewed all images. If necessary, OCT (Spectralis OCT, Heidelberg Engineering, Heidelberg, Germany) or fundus fluorescein angiography was performed to confirm DR severity. DR severity was classified according to the International Clinical Diabetic Retinopathy Severity Scales (ICDRSS) as follows (8): 0, no DR; 1, mild non-proliferative DR (NPDR); 2, moderate NPDR; 3, severe NPDR; 4, proliferative DR (PDR); and 5, ungradable image quality. When the severity differed between the two eyes, the worse eye determined the overall DR stage. In this study, RDR was defined as moderate NPDR or worse (ICDRSS grades 2–4), including the presence of diabetic macular edema (DME).

### Machine learning model development

In this study, the iDMas-DR framework (RetiMark, South Korea) was developed to detect RDR using systemic clinical and laboratory data. The dataset was split into training (30%) and validation (70%) subsets using random sampling by Python script. Four ML algorithms, logistic regression (LR), decision tree (DT), extreme gradient boosting (XGB), and random forest (RF), were trained on the training dataset. Their performance was compared using the validation dataset to select the most suitable model for further analysis. Among the four models, the RF model showed the highest area under the receiver operating characteristic curve (AUROC) and was selected as the final model.

Model training procedure followed three main steps: preprocessing, data preparation, and model training. In the preprocessing step, raw clinical data were cleaned by addressing missing values, outliers, and inconsistencies and removing duplicates. Categorical variables were encoded, and numerical variables were scaled using StandardScaler. In the data preparation step, class imbalance was corrected using the Synthetic Minority Over-sampling Technique (SMOTE). In the model training step, feature selection was performed using recursive feature elimination with cross-validation (RFE-CV). For the selected RF model, hyperparameters were optimized using RandomizedSearchCV with five-fold cross-validation. Tuned parameters included the number of trees, maximum tree depth, minimum samples required for node splits and leaf nodes, number of features considered per split, and the splitting criterion.

### Statistical analysis

All statistical analyses were performed using IBM SPSS Statistics (version 28.0) and Python (version 3.12.2). Continuous variables were compared using independent t-tests, and categorical variables were analyzed using the chi-squared or Fisher’s exact test, as appropriate. ML performance for detecting RDR was evaluated on the internal validation set using AUROC, recall (sensitivity), specificity, accuracy, precision (positive predictive value; PPV), negative predictive value (NPV), and F1-score, with 95% CI. The F1-score was additionally used to summarize the balance between precision and recall. Shapley Additive Explanations (SHAP), a model interpretation method based on game theory, was used for model interpretation and identification of key features for predicting RDR. A confusion matrix was used to assess classification results, and predicted probabilities with a probability threshold of 0.38 were visualized using boxplots. This threshold was chosen to balance sensitivity and specificity based on the distribution of predicted probabilities in the validation dataset. Multivariable logistic regression was performed using the total dataset (n = 562) to identify systemic predictors of RDR, defined either by a retinal specialist or the ML model (threshold ≥ 0.38).

## Results

### Baseline characteristics

**Figure 1:**
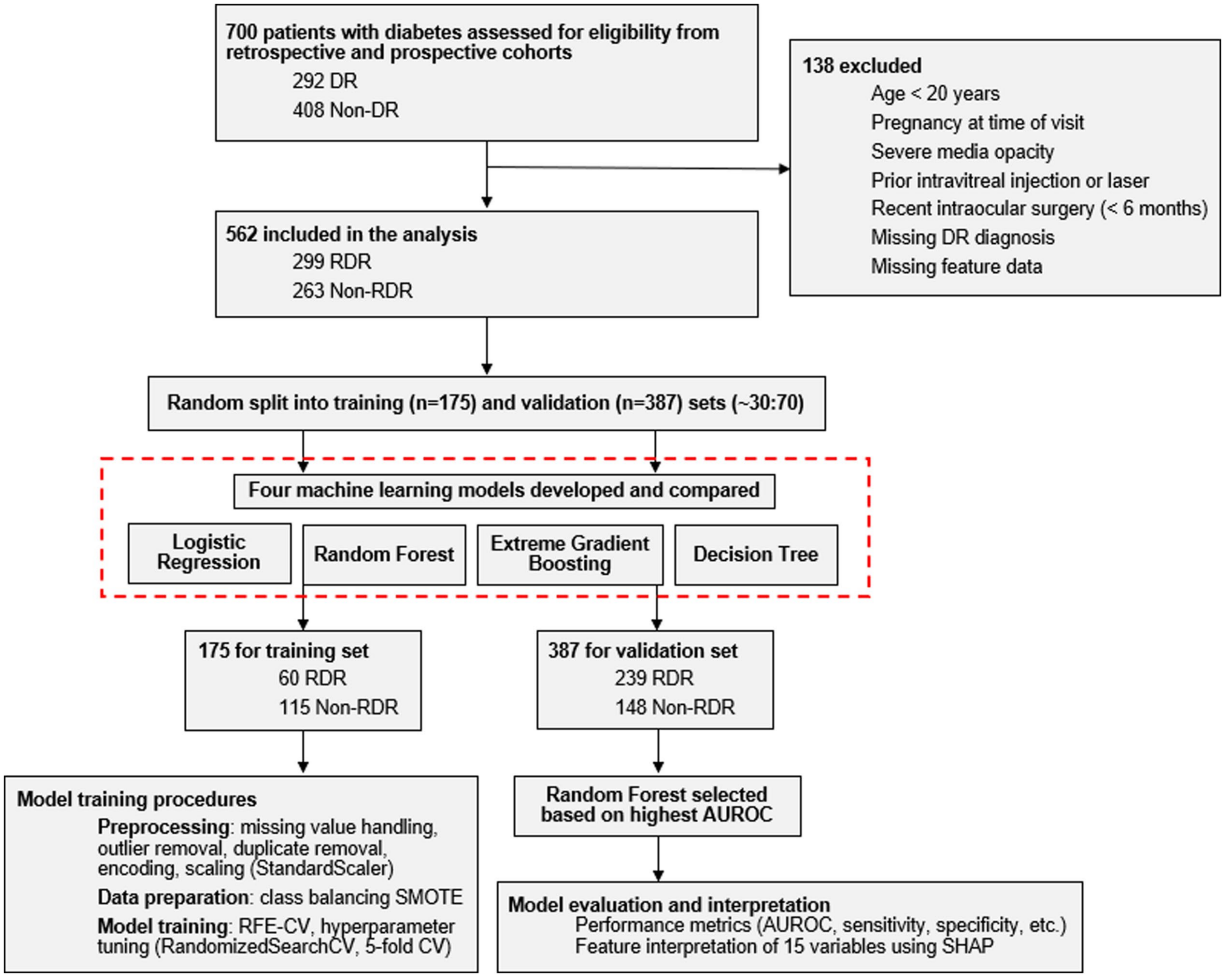
Flowchart showing patient selection and dataset division, and machine learning model development. DR, diabetic retinopathy; RDR, referable diabetic retinopathy; SHAP, Shapley additive explanations; AUROC, area under the receiver operating characteristic curve; CV, cross-validation.

The final dataset included 562 patients (299 with RDR, 263 with non-RDR) (Figure 1). Table 1 summarizes the baseline clinical characteristics of the training (*n* = 175) and validation (*n* = 387) datasets. Patients with RDR were significantly younger, exhibited higher fasting glucose levels and had longer diabetes duration and higher SBP (*p <* 0.05). In the validation dataset (Table 1) and total dataset (*n* = 562; Supplementary Table S1), RDR patients also had higher HbA_1c_ levels and lower eGFR (*p* < 0.05), though both were not statistically significant in the training dataset. DR severity staging for both training and validation datasets showed similar distributions.

**Table 1:** Baseline characteristics and DR severity in training and validation cohorts.

Total (*n* = 562)						
Characteristic	Train cohort (*n* = 175)			Validation cohort (*n* = 387)		
	RDR (*n* = 60)	Non-RDR (*n* = 115)	*P* value^a^	RDR (*n* = 239)	Non-RDR (*n* = 148)	*P* value^a^
Age at diabetes diagnosis, yr						
Mean (SD)	57.8 (9.9)	62.2 (10.0)	0.007	57.6 (10.8)	63.6 (10.4)	<0.0001
Sex, No. (%)						
Female	21 (35)	42 (36.5)	0.870	59 (24.7)	93 (62.8)	<0.001
Male	39 (65)	73 (63.5)		180 (75.3)	55 (37.2)	
Height, cm						
Mean (SD)	166.0 (8.9)	163.2 (8.9)	0.044	166.3 (8.2)	161.7 (9.1)	< 0.0001
Weight, kg						
Mean (SD)	67.7 (12.6)	67.7 (12.0)	0.999	68.1 (11.7)	64.7 (13.1)	0.0078
Body mass index, kg/m^2^						
Mean (SD)	24.4 (3.0)	25.3 (3.3)	0.078	24.5 (3.1)	24.6 (3.2)	0.913
Systolic blood pressure, mmHg						
Mean (SD)	136.0 (16.3)	136.4 (14.0)	0.869	133.2 (15.1)	135.8 (14.7)	0.108
Diastolic blood pressure, mmHg						
Mean (SD)	80.0 (9.6)	80.0 (14.8)	0.974	77.9 (9.9)	76.8 (10.7)	0.321
Duration of diabetes, yr						
Mean (SD)	14 (9.8)	11 (7.0)	0.041	15 (9.6)	12 (6.6)	0.0003
Diabetes type, No.						
Type 1 DM	0	1	NA	0	2	NA
Type 2 DM	60	114	NA	239	146	NA
Total cholesterol, mg/dL						
Mean (SD)	155 (39.3)	153 (32.2)	0.688	159 (42.7)	161 (37.9)	0.643
Fasting glucose, mg/dL						
Mean (SD)	168 (70.4)	149 (47.1)	0.031	154 (57.2)	138 (28.5)	0.002
HbA_1c_, %						
Mean (SD)	7.97 (1.6)	7.81 (1.2)	0.461	8.00 (1.5)	7.15 (0.9)	<0.001
eGFR, mL/min/1.73m^2^						
Mean (SD)	83.1 (27.1)	86.9 (21.6)	0.322	78.2 (26.9)	89.7 (15.1)	< 0.001
Cystatin C, mg/L						
Mean (SD)	0.94 (0.45)	0.90 (0.55)	0.646	0.99 (0.47)	0.76 (0.16)	< 0.001
Insulin treatment, No. (%)						
Yes	13 (21.7)	28 (24.3)	0.851	72 (30.1)	16 (10.8)	<0.001
Smoking status, No. (%)						
Non-smoker	30 (50)	74 (64.3)	0.183	105 (43.9)	119 (80.4)	<0.001
Ex-smoker	16 (26.7)	21 (18.3)		79 (33.1)	15 (10.1)	
Current smoker	14 (23.3)	20 (17.4)		55 (23.0)	14 (9.5)	
DR stage, No. (%)^b^						
*Non-RDR*						
No DR	NA	95 (54.3)	NA	NA	131 (33.9)	NA
Mild NPDR	NA	20 (11.4)	NA	NA	17 (4.4)	NA
*RDR*						
Moderate NPDR	11 (6.3)	NA	NA	61 (15.8)	NA	NA
Severe NPDR	20 (11.4)	NA	NA	52 (13.4)	NA	NA
PDR	29 (16.6)	NA	NA	126 (32.6)	NA	NA
DME	4 (2.3)	NA	NA	9 (2.3)	NA	NA

Values are presented as mean (SD) or number (%).

NA, not applicable; HbA_1c,_ hemoglobin A_1c_; eGFR, estimated glomerular filtration rate; DR, diabetic retinopathy; RDR, referable diabetic retinopathy; PDR, proliferative diabetic retinopathy; DME, diabetic macular edema; DM, diabetes mellitus.

a *P* value for the difference between the train and validation sets, using a two-tailed *t*-test.

b The grading of DR was done by an ophthalmologist.

### Model performance

**Figure 2:**
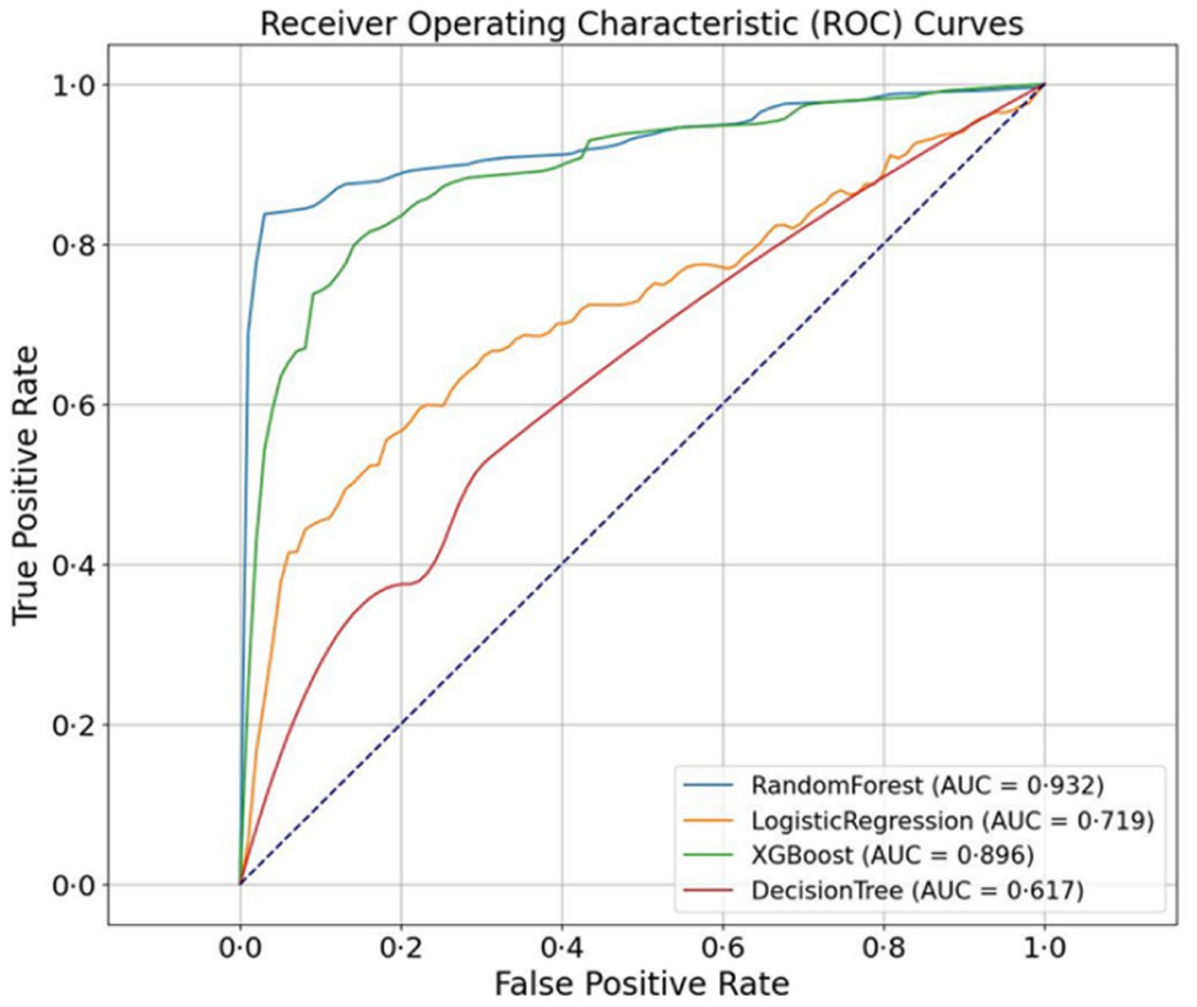
Receiver operating characteristic curves comparing the four machine learning models. The random forest model achieved the highest AUC (0.932). AUC, area under the curve.

All four MLs were trained on the same dataset (*n* = 175) to detect RDR. Using the internal validation dataset (*n* = 387), the RF model demonstrated the highest performance (AUROC 0.932), followed by XGB (0.896), LR (0.719), and DT (0.617) (Figure 2). The RF model was selected as the final model and showed an AUROC of 0.932, sensitivity of 0.858, specificity of 0.912, accuracy of 0.879, PPV of 0.940, and NPV of 0.799, with an odds ratio of 62.6 (*p* < 0.001 by chi-squared test), compared with retina specialist grading (Table 2). Performance remained consistent at a 0.38 threshold, with an F1-score of 0.897 (Table 3). Predicted probability distributions are shown in boxplots (Supplementary Figure S1), and the results, including sensitivity, specificity for various thresholds are presented in Supplementary Table S6.

**Table 2:** Confusion matrix of the RF model for RDR classification in the validation cohort (*n* = 387).

Reference^a^	Predicted RDR, *n*	Predicted non-RDR, *n*	Total, *n*
RDR^b^ (*n* = 239)	205	34	239
Non-RDR (*n* = 148)	13	135	148
Total	218	169	387

Values are presented as number.

RF, random forest; DR, diabetic retinopathy; RDR, referable diabetic retinopathy.

a Reference grading by retinal specialists.

b RDR was defined as moderate non-proliferative, severe non-proliferative, or proliferative diabetic retinopathy, including diabetic macular edema.

**Table 3:** Performance metrics of the RF model for RDR classification in the validation cohort (*n* = 387).

Metric	Value (95% CI)
AUROC (95% CI)	0.932 (0.904–0.955)
Sensitivity (95% CI)	0.858 (0.823–0.893)
Specificity (95% CI)	0.912 (0.867–0.958)
Accuracy (95% CI)	0.879 (0.846–0.911)
PPV (95% CI)	0.940 (0.917–0.964)
NPV (95% CI)	0.799 (0.738–0.859)
F1-score^a^	0.897

Values are presented as number (95% CI).

RF, random forest; RDR, referable diabetic retinopathy; AUROC, area under the ROC curve; PPV, positive predictive value; NPV, negative predictive value; CI, confidence interval.

a Calculated as 2 × (precision × recall) / (precision + recall), using the PPV (precision) and sensitivity (recall).

### Predictor importance by SHAP

**Figure 3:**
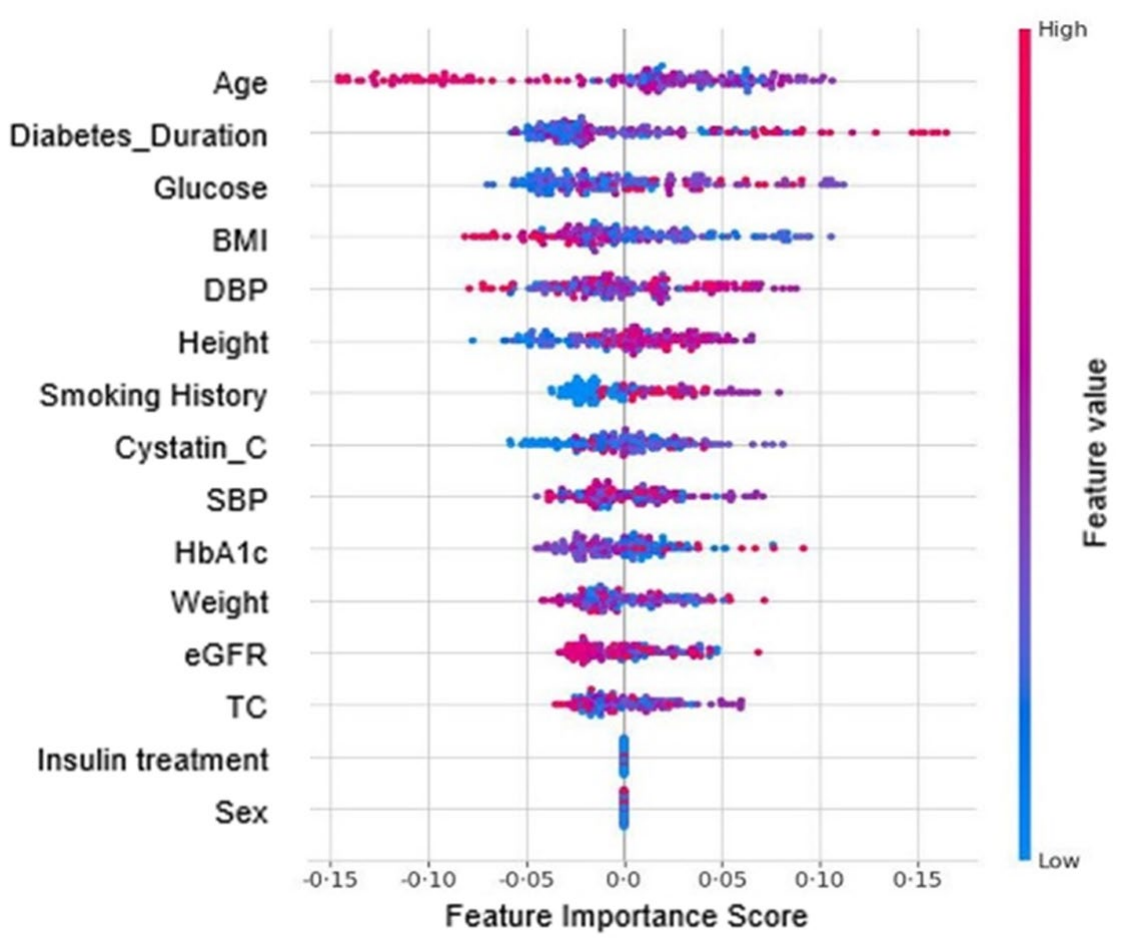
Summary plot of Shapley additive explanations (SHAP) showing feature importance contributing to referable diabetic retinopathy (RDR) prediction by the random forest model.

Fifteen clinical variables were selected through RFE-CV during model training: age, sex, height, weight, systolic and diastolic blood pressure, duration of diabetes, total cholesterol, fasting glucose, HbA_1c_, eGFR, Cystatin C, body mass index (BMI), insulin use, and smoking status. Predictor importance for the RF model was interpreted using SHAP analysis (Figure 3), based on variables selected through RFE-CV. Age, diabetes duration, fasting glucose, BMI and DBP were the most significant predictors, reflecting known risk factors for DR such as prolonged diabetes duration, poor glycemic control, and coexisting nephropathy (9–17). The top eight features with specific mean SHAP values for each model are shown in the Supplementary Table S2. In the RF model, age had the greatest inverse impact on RDR detection, followed by diabetes duration, glucose, BMI, DBP, height, smoking history, Cystatin C, SBP, HbA_1c_, weight, eGFR, and total cholesterol. Insulin use and sex showed comparatively lower SHAP values in the RF model. Results for the other three ML models are provided in the Supplementary Figure S2.

### Sequential feature inclusion

Model Performance improved with inclusion of variables step-by-step. AUROC increased from 0.78 (using age, diabetes duration, glucose, and BMI) to 0.932 with all 15 features, suggesting that each contributes to RDR prediction (Supplementary Table S3).

### Comparison with regression

We assessed variance inflation factors to address multicollinearity. Weight and height were excluded due to high collinearity, while BMI retaining as a composite indicator. Age, diabetes duration, and eGFR remained significantly associated with RDR in both the human and ML models (Supplementary Table S4). HbA_1c_ was significant only in the human-based model, while BMI became significant in both after adjustment. Glucose, DBP, and Cystatin C showed high SHAP values but were not statistically significant in regression, reflecting differences between model-based importance and independent association.

### Prevalence-based performance analysis

We simulated model performance under varying RDR prevalence assumptions ranging from 15 to 60%. As prevalence increased (15–60%), PPV rose from 63.3 to 93.6%, while NPV declined from 97.3 to 81.0%, highlighting the need to consider local DR prevalence when applying the model (Supplementary Table S5).

## Discussion

In this study, we developed and validated a RF-based ML model, iDMas-DR, to predict RDR using only systemic clinical and laboratory data. The model achieved high diagnostic performance (AUROC 0.932) with balanced sensitivity, specificity, and F1-score, supporting its potential utility in settings without retinal imaging infrastructure.

Several studies using only clinical data for DR screening have reported relatively lower sensitivity or specificity for DR detection compared to image-based models (18–22). A US-based study using electronic medical records showed modest performance (AUROC 0.72, sensitivity 69.2%, specificity 55.9%) (23). A LASSO-based model using data from the Korea National Health and Nutrition Examination Surveys (KNHANES) achieved sensitivity of 72.1% and specificity of 76.0% (*n* = 562) (19). A support vector machine model (20) reported 93% sensitivity but only 72% specificity (*n* = 536). Deep neural network (DNN) models using clinical data have reported sensitivities of 72.2 and 76.0%, with specificities of 74.2 and 80.4%, respectively (21, 22). The former study used basic clinical parameters such as BMI, diabetes duration, age, blood glucose levels, and blood pressure (*n* = 175) (21), whereas the latter included a broader set of variables, including comorbidities, treatment history, and lifestyle factors (*n* = 536) (22). More recent study using a RF model with broader clinical data (e.g., variables such as age, gender, diabetes type, treatment history, DM control status, family history, pregnancy history, and systemic comorbidities), reported accuracy of 76.0%, sensitivity of 53.0%, and specificity of 80.0% for detecting sight-threatening diabetic retinopathy (STDR) (18). These models used limited input variables and often lacked interpretability. In contrast, our model incorporates a broader range of clinical features, including glycemic, renal, and anthropometric markers. A comparison of our model with traditional clinical risk scores and prior ML-based studies is provided in Table 4.

**Table 4:** Comparison of clinical data–based models for diabetic retinopathy detection.

Study	Model	Sample size (*n*)	Target outcome	AUROC	Sensitivity	Specificity
Hosseini et al. (36)	Logistic regression	3,734	DR	0.70	60.3	69.4
Aspelund et al. (37)	Risk assessment algorithm	5,199	STDR	0.76	NR	NR
Semeraro et al. (38)	Cox’s proportional hazard model	5,034	DR	0.75 (C-index) 0.68 (Gonen-Heller CPE)	NR	NR
Ogunyemi et al. (23)	RUSBoost	513	DR	0.72	69.2	55.9
Oh et al. (19)	LASSO	327	DR	0.81	77.4	72.7
Tsao et al. (39)	SVM	536	DR	0.84	78.7	66.4
Alfian et al. (21)	DNN + RFE	133	DR	0.80	76.0	80.4
Ogunyemi et al. (22)	DNN	40,631	DR	0.80	72.2	74.2
Venkatesh et al. (18)	RF	1,416	STDR	NR	53.0	80.0
Present study	RF	562	RDR	0.93	85.8	91.2

AUROC, area under the receiver operating characteristic curve; DNN, deep neural networks; DR, diabetic retinopathy; NR, not reported, RDR, referable diabetic retinopathy; RF, random forest model; RFE, recursive feature elimination; STDR, sight-threatening diabetic retinopathy; SVM, support vector machines.

By comparison, fundus image-based AI models have demonstrated excellent diagnostic performance, with AUROC up to 0.97 (1, 24, 25). A deep learning model developed by Google Research achieved high diagnostic accuracy (26). The IDx-DR system, the first FDA-approved AI-based DR screening tool (27), also showed high performance in primary care. A meta-analysis of deep learning models reported pooled AUROC of 0.97, with sensitivity of 0.83 and specificity of 0.92 (24). However, image-based systems require specialized infrastructure and trained personnel, which can limit adoption in many real-world or resource-limited settings. Further, challenges such as poor pupil dilation, image quality, and patient compliance may reduce real-world utility (1, 25).

In comparison, our clinical-only model does not rely on retinal imaging, making it more accessible in resource-limited settings. We selected the RF algorithm for its reliable performance and stability in handling structured clinical data (28). Tree-based models such as RF, XGB, and CatBoost have gained popularity in clinical prediction tasks due to their robustness and ability to handle routine health records (29). More recently, models such as LightGBM and TabNet have been developed to improve performance on structured clinical data, while DNN offer greater flexibility for diverse data types but often lack interpretability and require larger datasets (26). The RF model remains a practical and competitive choice due to its balance of performance, interpretability, and ease of use. To improve model performance, key hyperparameters were tuned, and 15 predictors were selected using RFE-CV, a performance-driven feature selection method. Clinically relevant variables, such as comorbidities and medication history, excluding insulin use, were not included in the final model due to incomplete or non-standardized data reporting, which could have introduced bias. Because RFE-CV does not offer interpretable variable importance, we applied SHAP to estimate each feature’s contribution to individual predictions. SHAP revealed clinically relevant predictors and showed complex, non-linear relationships that were not evident in multivariable regression, supporting model transparency (30). For instance, glucose, DBP, and Cystatin C had high SHAP values but were not statistically significant in regression (Supplementary Table S4). SHAP considers the impact of each feature within the full model context, while regression estimates the independent effect of each variable (30).

Among the SHAP-ranked features, Cystatin C, a more sensitive marker of early renal impairment than eGFR (14, 31), reflects systemic microvascular damage such as diabetic nephropathy and may help explain its association with retinopathy. It serves as a practical alternative to urine-based testing when evaluating DR risk, allowing insight into renal function without requiring urine collection. Patients with RDR were significantly younger than those without RDR (mean age 57.6 vs. 63.6 years; *p* < 0.001; Table 1), consistent with prior findings that earlier-onset diabetes increases DR risk (32). Although sex and insulin use had low SHAP values and were not statistically significant (Table 1; Figure 3), both showed significant differences in the total dataset (*n* = 562; Supplementary Table S1) and were retained due to their clinical relevance. Notably, in tree-based models, variables with low individual contribution may still support prediction through interaction effects (29).

Our study has several strengths. First, the proposed model can be readily integrated into CDSS in primary care or endocrinology settings, as it relies solely on routinely collected clinical and laboratory data. This approach on non-imaging data make the model especially suitable for environments where ophthalmic imaging may not be accessible, although the inclusion of ocular parameters such as biometry or choroidal thickness could potentially provide additional predictive value (33, 34). By automating risk assessments, this approach could significantly enhance referral efficiency, enabling timely identification of RDR without the need for specialized equipment. In addition, broader application in workplace or community-based screening programs may help reduce vision-threatening DR at the population level. Further, integrating this model into electronic medical record systems could enable real-time monitoring and dynamic decision-making, improving patient outcomes across various clinical settings.

This study also has limitations. First, it was based on a single-center dataset from a tertiary hospital, potentially introducing selection bias. Second, the inclusion of both retrospective and prospective cohorts may have caused heterogeneity. Third, DR grading was performed by a single experienced retina specialist without independent validation or assessment of interobserver agreement, which may introduce some misclassification bias. Fourth, DR severity was determined using OCT or fundus fluorescein angiography as supplementary tools, without a fully standardized protocol. A more consistent approach for assessing DR severity and DME would likely improve reproducibility and reduce potential diagnostic variability. Fifth, all participants were of Korean ethnicity, which may limit the applicability of the model to other ethnic or geographic populations, and thus external validation in other populations is required. Finally, due to its retrospective design, additional prospective real-world validation is needed before widespread clinical implementation. While these limitations are acknowledged, we have performed initial validation using the KNHANES data, which has significant missing data, and limits its ability to fully assess the model’s performance (35). To address this, a large-scale multicenter external validation is planned to further validate the model’s effectiveness in a more comprehensive and diverse population.

In conclusion, we present a clinically interpretable ML model that predicts RDR using routine clinical data. The model achieved strong predictive performance without the use of imaging. As an accessible tool, it could support early DR detection, reduce unnecessary referrals, and help prevent vision loss in low-resource settings.

## Data Availability

The raw data supporting the conclusions of this article will be made available by the authors, without undue reservation.
