# Supplementary material for: Detection of referable diabetic retinopathy using machine learning on routine clinical data
Source: Front Med (Lausanne). 2026 May 8;13:1807809. doi: 10.3389/fmed.2026.1807809 (PMC13227111; doi:10.3389/fmed.2026.1807809)
Supplement: Supplementary file 1 [file Supplementary_file_1.DOCX]

**List of supplementary Tables and Figures**

**Supplementary Table 1.** Baseline clinical characteristics of the total dataset (n = 562)

**Supplementary Table 2.** The Shapley Additive Explanations (SHAP) value summary of top-ranked features in four machine learning models

**Supplementary Table 3.** Stepwise performance metrics by sequential addition of clinical features for validation sets (n=387)

**Supplementary Table 4.** Multivariable regression comparing clinical predictors of referable diabetic retinopathy (RDR): human vs. machine learning (ML) grading (n=562)

**Supplementary Table 5.** Predicted metrics of random forest (RF) model at varying referable diabetic retinopathy (RDR) prevalence (15-60%), on the validation dataset (n=387)

**Supplementary Table 6.** Performance metrics of the random forest (RF) model for referable diabetic retinopathy (RDR) classification at different thresholds

**Supplementary Fig. 1.** Boxplot of predicted probabilities for referable diabetic retinopathy (RDR) vs. non-RDR cases

**Supplementary Fig. 2.** Shapley Additive Explanations (SHAP) feature importance plots for (A) logistic regression (LR), (B) extreme gradient boosting (XGB), and (C) decision tree (DT) models

**Supplementary Table 1.** Baseline clinical characteristics of the total dataset (n = 562)

| Characteristic | RDR (n=299) | Non-RDR (n=263) | *P* value^a^ |
| --- | --- | --- | --- |
| Age at diabetes diagnosis, yr |  |  |  |
| Mean (SD) | 57.7 (10.6) | 63.0 (10.2) | < 0.0001 |
| Sex, No. (%) |  |  |  |
| Male | 219 (73.2) | 128 (48.7) | < 0.0001 |
| Female | 80 (26.8) | 135 (51.3) |  |
| Height, cm |  |  |  |
| Mean (SD) | 166.3 (8.3) | 162.3 (9.0) | < 0.0001 |
| Weight, kg |  |  |  |
| Mean (SD) | 68.0 (11.8) | 66.0 (12.7) | 0.0537 |
| Body mass index, kg/m^2^ |  |  |  |
| Mean (SD) | 24.5 (3.1) | 24.9 (3.3) | 0.1471 |
| Systolic blood pressure, mmHg |  |  |  |
| Mean (SD) | 133.8 (15.4) | 136.0 (14.4) | 0.0743 |
| Diastolic blood pressure, mmHg |  |  |  |
| Mean (SD) | 78.3 (9.9) | 78.2 (12.7) | 0.9016 |
| Duration of diabetes, yr |  |  |  |
| Mean (SD) | 14.6 (9.6) | 11.5 (6.8) | < 0.0001 |
| Diabetes type, n |  |  |  |
| Type 1 | 0 | 3 | NA |
| Type 2 | 299 | 260 | NA |
| Total cholesterol, mg/dL |  |  |  |
| Mean (SD) | 158.3 (42.0) | 157.6 (35.7) | 0.8199 |
| Fasting glucose, mg/dL |  |  |  |
| Mean (SD) | 156.4 (60.2) | 142.5 (38.1) | 0.0010 |
| HbA_1c_, (%) |  |  |  |
| Mean (SD) | 8.0 (1.5) | 7.4 (1.1) | < 0.0001 |
| eGFR, mL/min/1.73m^2^ |  |  |  |
| Mean (SD) | 79.2 (27.0) | 88.5 (18.2) | < 0.0001 |
| Cystatin C, mg/L |  |  |  |
| Mean (SD) | 1.0 (0.5) | 0.8 (0.4) | < 0.0001 |
| Insulin treatment, No. (%) |  |  |  |
| Yes | 85 (28.4) | 44 (16.7) | 0.0014 |
| Smoking status, No. (%) |  |  |  |
| Non-smoker | 135 (45.2) | 193 (73.4) | < 0.0001 |
| Ex smoker | 95 (31.8) | 35 (13.3) | < 0.0001 |
| Current smoker | 69 (23.1) | 35 (13.3) | < 0.0001 |
| DR stage, No. (%)^b^ |  |  |  |
| Non-RDR |  |  |  |
| No DR | NA | 226 (85.9) | NA |
| Mild NPDR | NA | 37 (14.1) | NA |
| RDR |  |  |  |
| Moderate NPDR | 72 (24.1) | NA | NA |
| Severe NPDR | 72 (24.1) | NA | NA |
| PDR | 155 (51.8) | NA | NA |
| DME | 13 (4.3) | NA | NA |

^a^P value for the difference between the train and validation sets, using a two-tailed t-test.

^b^The grading of DR was done by an ophthalmologist.

NA, not applicable; HbA_1c_, hemoglobin A_1c_; eGFR, estimated glomerular filtration rate; DR, diabetic retinopathy; RDR, referable diabetic retinopathy; PDR, proliferative diabetic retinopathy; DME, diabetic macular edema; DM, diabetes mellitus.

**Supplementary Table 2.** The Shapley Additive Explanations (SHAP) value summary of top-ranked features in four machine learning models

| Features | SHAP value | Effects |
| --- | --- | --- |
| Logistic Regression |  |  |
| Age | 0.0591 | 0.1923 |
| Insulin treatment | 0.0588 | 0.1915 |
| BMI | 0.0518 | 0.1687 |
| Diabetes duration | 0.0460 | 0.1497 |
| eGFR | 0.0372 | 0.1210 |
| Height | 0.0338 | 0.1101 |
| Cystatin C | 0.0144 | 0.0468 |
| Weight | 0.0061 | 0.0199 |
| Extreme gradient boosting |  |  |
| Age | 0.1081 | 0.2731 |
| eGFR | 0.0417 | 0.1054 |
| Diabetes duration | 0.0391 | 0.0988 |
| BMI | 0.0323 | 0.0817 |
| DBP | 0.0242 | 0.0612 |
| Cystatin C | 0.0230 | 0.0581 |
| Glucose | 0.0207 | 0.0523 |
| SBP | 0.0202 | 0.0510 |
| Weight | 0.0175 | 0.0442 |
| Height | 0.0167 | 0.0422 |
| Smoking history | 0.0149 | 0.0377 |
| Insulin treatment | 0.0135 | 0.0342 |
| Total cholesterol | 0.0129 | 0.0325 |
| HbA_1c_ | 0.0110 | 0.0278 |
| Decision Tree |  |  |
| Age | 0.1205 | 0.2273 |
| BMI | 0.0812 | 0.1532 |
| SBP | 0.0699 | 0.1317 |
| Cystatin C | 0.0626 | 0.1181 |
| Height | 0.0462 | 0.0872 |
| Diabetes duration | 0.0451 | 0.0850 |
| Total cholesterol | 0.0435 | 0.0820 |
| Smoking history | 0.0400 | 0.0754 |
| eGFR | 0.0213 | 0.0401 |
| Random forest |  |  |
| Age | 0.0554 | 0.1604 |
| Diabetes duration | 0.0387 | 0.1120 |
| Glucose | 0.0341 | 0.0986 |
| BMI | 0.0314 | 0.0908 |
| DBP | 0.0287 | 0.0830 |
| Height | 0.0253 | 0.0731 |
| Smoking history | 0.0211 | 0.0609 |
| Cystatin C | 0.0207 | 0.0599 |
| SBP | 0.0189 | 0.0547 |
| HbA_1c_ | 0.0185 | 0.0536 |
| Weight | 0.0184 | 0.0533 |
| eGFR | 0.0178 | 0.0516 |
| Total cholesterol | 0.0166 | 0.0482 |

Shapley Additive Explanations, SHAP; BMI, body mass index; SBP, systolic blood pressure; DBP, diastolic blood pressure; HbA_1c_, glycosylated hemoglobin; DM, diabetes mellitus; eGFR, estimated glomerular filtration rate.

**Supplementary Table 3.** Stepwise performance metrics by sequential addition of clinical features for validation sets (n=387)

| Features | AUROC (95% CI) | Sensitivity (95% CI) | Specificity (95% CI) | Accuracy (95% CI) |
| --- | --- | --- | --- | --- |
| Age, DM duration, Glucose, BMI | 0.789 (0.739-0.830) | 0.753 (0.710-0.796) | 0.669 (0.593-0.744) | 0.721 (0.676-0.766) |
| Age, DBP, DM duration, Glucose, BMI | 0.825 (0.778-0.862) | 0.770 (0.728-0.811) | 0.676 (0.600-0.751) | 0.734 (0.689-0.777) |
| Age, Height, DBP, DM duration, Glucose, BMI | 0.848 (0.806-0.884) | 0.762 (0.719-0.803) | 0.770 (0.702-0.838) | 0.765 (0.722-0.807) |
| Age, Height, DBP, DM duration, Glucose, HbA_1c_, BMI, Smoking | 0.848 (0.806-0.884) | 0.762 (0.719-0.803) | 0.770 (0.702-0.838) | 0.765 (0.722-0.807) |
| Age, Height, DBP, DM duration, Glucose, Cystatin C, BMI, Smoking | 0.871 (0.830-0.904) | 0.782 (0.741-0.823) | 0.824 (0.763-0.885) | 0.798 (0.758-0.838) |
| Age, Height, SBP, DBP, DM duration, Glucose, Cystatin C, BMI, Smoking | 0.889 (0.854-0.919) | 0.803 (0.763-0.842) | 0.811 (0.748-0.873) | 0.806 (0.767-0.846) |
| Age, Height, SBP, DBP, DM duration, Glucose, HbA_1c_, Cystatin C, BMI, Smoking | 0.883 (0.847-0.914) | 0.787 (0.746-0.828) | 0.818 (0.756-0.879) | 0.798 (0.759-0.839) |
| Age, Height, Weight, SBP, DBP, DM duration, Glucose, HbA_1c_, Cystatin C, BMI, Smoking | 0.880 (0.847-0.910) | 0.766 (0.723-0.808) | 0.791 (0.724-0.857) | 0.775 (0.733-0.817) |
| Age, Height, Weight, SBP, DBP, DM duration, Glucose, HbA_1c_, eGFR, Cystatin C, BMI, Smoking | 0.904 (0.874-0.932) | 0.803 (0.763-0.842) | 0.892 (0.841-0.941) | 0.837 (0.800-0.873) |
| Age, Height, Weight, SBP, DBP, DM duration, Total cholesterol, Glucose, HbA_1c_, eGFR, Cystatin C, BMI, Smoking | 0.916 (0.886-0.942) | 0.820 (0.781-0.859) | 0.878 (0.826-0.931) | 0.842 (0.807-0.879) |
| Age, Height, Weight, SBP, DBP, DM duration, Total cholesterol, Glucose, HbA_1c_, eGFR, Cystatin C, BMI, Insulin, Smoking | 0.893 (0.859-0.922) | 0.766 (0.723-0.808) | 0.878 (0.826-0.931) | 0.809 (0.769-0.848) |
| Age, Height, Weight, SBP, DBP, DM duration, Total cholesterol, Glucose, HbA_1c_, eGFR, Cystatin C, BMI, Sex, Insulin, Smoking | 0.932 (0.904-0.955) | 0.858 (0.823-0.893) | 0.912 (0.867-0.958) | 0.879 (0.846-0.911) |

AUROC, area under the receiver operating characteristic curve; DM, diabetes mellitus; eGFR, estimated glomerular filtration rate; BMI, body mass index; DBP, diastolic blood pressure; HbA_1c_, hemoglobin A_1c_; SBP, systolic blood pressure.

**Supplementary Table 4.** Multivariable regression comparing clinical predictors of referable diabetic retinopathy (RDR): human vs. machine learning (ML) grading (n=562)

|  | Reference grading by a retinal specialist |  | Machine learning predicted classification |  | Group difference |  |
| --- | --- | --- | --- | --- | --- | --- |
| Variable | OR (95% CI) | *P* value^a^ | OR (95% CI) | *P* value^a^ | Difference in standardized ORs (95% CI) | *P* value^a^ |
| Age (per 1-year increase) | 0.90 (0.88–0.92) | 0.0000 | 0.91 (0.89–0.94) | 0.0000 | 0.015 (-0.022–0.052) | 0.4364 |
| Duration of diabetes (per 1-year increase) | 1.08 (1.05–1.11) | 0.0000 | 1.07 (1.04–1.10) | 0.0000 | -0.015 (-0.054–0.025) | 0.4650 |
| Fasting glucose (per 1 mg/dL increase) | 1.00 (1.00–1.01) | 0.6524 | 1.00 (1.00–1.00) | 0.9685 | -0.001 (-0.008–0.006) | 0.7115 |
| BMI (kg/m²) | 0.84 (0.78–0.91) | 0.0000 | 0.85 (0.79–0.92) | 0.0000 | 0.013 (-0.089–0.116) | 0.7966 |
| Diastolic blood pressure (mmHg) | 1.00 (0.98–1.03) | 0.7489 | 1.02 (0.99–1.04) | 0.1436 | 0.014 (-0.021–0.048) | 0.4330 |
| Cystatin C (mg/L) | 0.93 (0.47–1.85) | 0.8366 | 0.68 (0.32–1.42) | 0.2984 | -0.320 (-1.329–0.689) | 0.5339 |
| Systolic blood pressure (mmHg) | 0.99 (0.97–1.01) | 0.2843 | 0.98 (0.96–1.00) | 0.0259 | -0.010 (-0.034–0.014) | 0.4104 |
| HbA₁c (per 1% increase) | 1.27 (1.04–1.55) | 0.0209 | 1.16 (0.97–1.39) | 0.1009 | -0.085 (-0.354–0.183) | 0.5343 |
| eGFR (mL/min/1.73m²) | 0.96 (0.95–0.98) | 0.0000 | 0.97 (0.95–0.98) | 0.0000 | 0.004 (-0.016–0.025) | 0.6876 |
| Total cholesterol (mg/dL) | 1.00 (1.00–1.01) | 0.6417 | 1.00 (1.00–1.01) | 0.2194 | 0.002 (-0.006–0.010) | 0.5991 |
| Ex-smoker (vs never smoker) | 3.45 (1.95–6.12) | 0.0000 | 3.33 (1.91–5.81) | 0.0000 | -0.034 (-0.832–0.764) | 0.9335 |
| Current smoker (vs never smoker) | 1.51 (0.82–2.79) | 0.1894 | 1.65 (0.91–2.99) | 0.1007 | 0.088 (-0.768–0.945) | 0.8404 |
| Insulin treatment (yes vs no) | 1.08 (0.64–1.82) | 0.7814 | 1.01 (0.61–1.68) | 0.9680 | -0.064 (-0.793–0.665) | 0.8640 |
| Female sex (vs male) | 0.55 (0.34–0.91) | 0.0209 | 0.51 (0.30–0.87) | 0.0144 | -0.081 (-0.816–0.654) | 0.8284 |

Predictions from the ML model were binarised at a probability threshold of 0.38.

^a^*P* value for the difference in the standardized ORs in the multivariable regression between the ML model and reference grading by a retinal specialist.

OR, odds ratios; CI, confidence intervals; BMI, body mass index; DBP, diastolic blood pressure; SBP, systolic blood pressure; HbA₁c, glycated hemoglobin; eGFR, estimated glomerular filtration rate.

**Supplementary Table 5.** Predicted metrics of random forest (RF) model at varying referable diabetic retinopathy (RDR) prevalence (15-60%), on the validation dataset (n=387)

| Data set | Prevalence of DR (%) | PPV, % (95% CI) | NPV, % (95% CI) | Accuracy, % (95% CI) |
| --- | --- | --- | --- | --- |
| Validation set (n=387) | 15 | 63.3 (50.6-74.4) | 97.3 (96.4-98.0) | 90.4 (87.0-93.2) |
|  | 20 | 52.0 (39.2-64.6) | 98.3 (97.7-98.8) | 90.1 (86.7-92.9) |
|  | 30 | 80.7 (71.3-87.6) | 93.7 (91.6-95.4) | 89.6 (86.1-92.4) |
|  | 40 | 86.7 (79.4-91.7) | 90.6 (87.5-93.0) | 89.0 (85.5-92.0) |
|  | 50 | 90.7 (85.3-94.3) | 86.5 (82.4-89.8) | 88.5 (84.9-91.5) |
|  | 60 | 93.6 (89.7-96.1) | 81.0 (75.7-85.4) | 88.0 (84.3-91.0) |

DR, diabetic retinopathy; PPV, positive predictive value; NPV, negative predictive value.

**Supplementary Table 6.** Performance metrics of the random forest (RF) model for referable diabetic retinopathy (RDR) classification at different thresholds

| Metric | Value | | | |
| --- | --- | --- | --- | --- |
| Threshold | 0.30 | 0.38 | 0.40 | 0.43 |
| AUROC | 0.932 | 0.932 | 0.932 | 0.932 |
| Sensitivity | 0.967 | 0.858 | 0.782 | 0.657 |
| Specificity | 0.351 | 0.912 | 0.993 | 1.000 |
| Accuracy | 0.731 | 0.879 | 0.863 | 0.788 |
| PPV | 0.706 | 0.940 | 0.995 | 1.000 |
| NPV | 0.867 | 0.799 | 0.739 | 0.643 |

AUROC, area under the ROC curve; PPV, positive predictive value; NPV, negative predictive value.

**Figures**

**Supplementary Fig. 1.** Boxplot of predicted probabilities for referable diabetic retinopathy (RDR) vs. non-RDR cases

The red line indicates the classification threshold (0.38).

RDR, referable diabetic retinopathy.

**
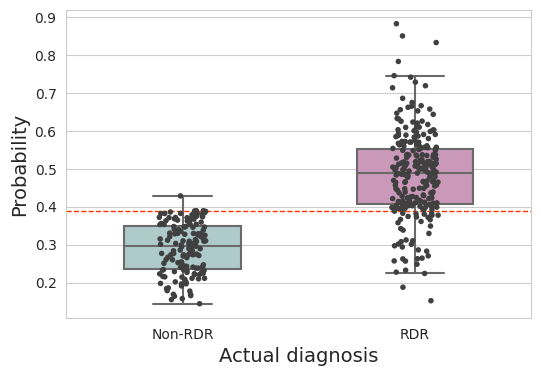
**

**Supplementary Fig. 2.** Shapley Additive Explanations (SHAP) feature importance plots for (A) logistic regression (LR), (B) extreme gradient boosting (XGB), and (C) decision tree (DT) models

Features are ordered by mean SHAP value. Color represents feature value (red = high, blue = low).

LR, logistic regression; XGB, extreme gradient boosting; DT, decision tree; BMI, body mass index; SBP, systolic blood pressure; DBP, diastolic blood pressure; HbA_1c_, glycosylated hemoglobin; DM, diabetes mellitus; eGFR, estimated glomerular filtration rate; TC, total cholesterol.


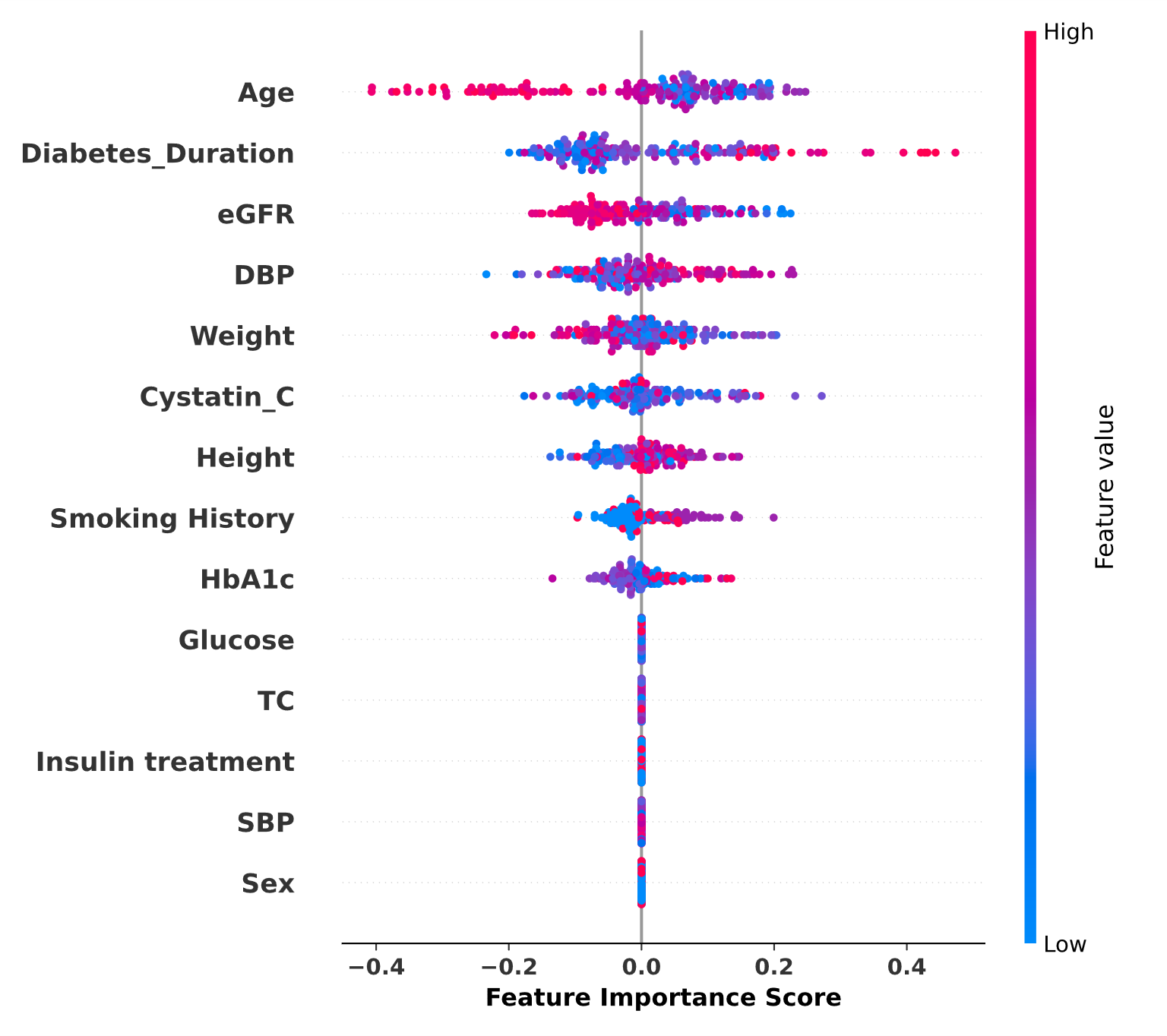


**(B) XGB Classifier**


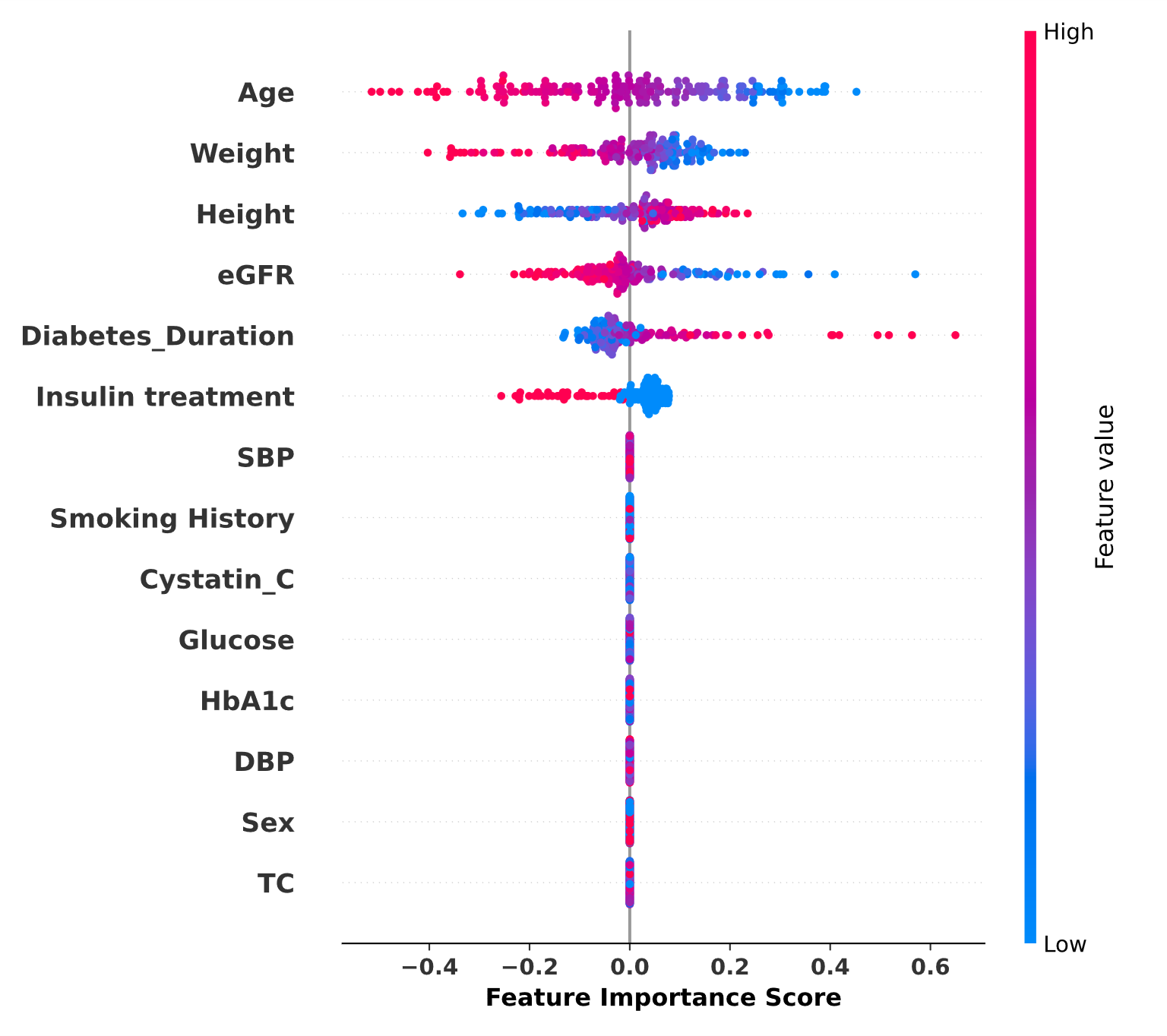


**(A) Logistic Regression**


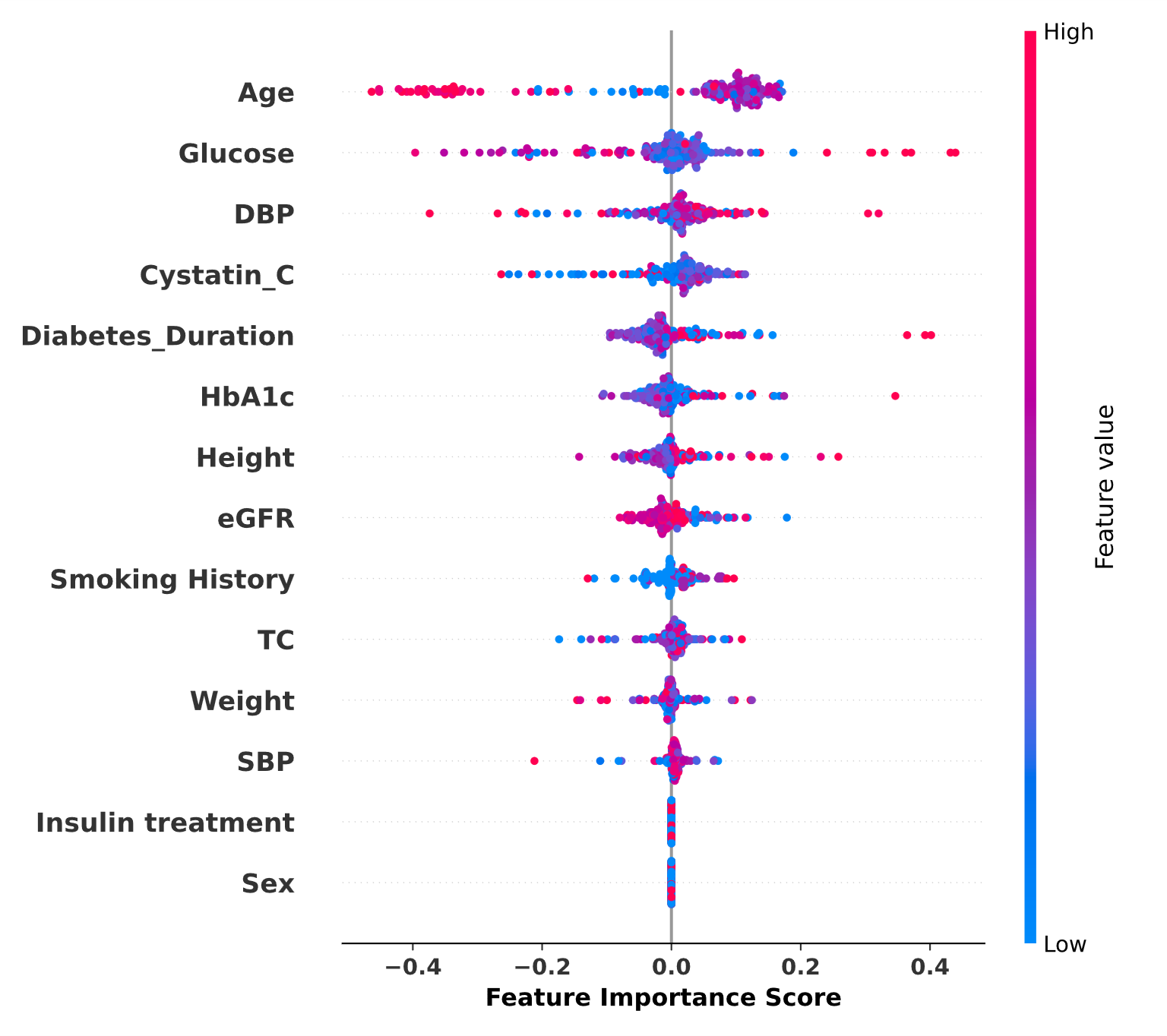


**(C) Decision Tree Classifier**
